# A Fault Diagnosis Scheme Using Hurst Exponent for Metal Particle Faults in GIL/GIS

**DOI:** 10.3390/s22030862

**Published:** 2022-01-23

**Authors:** Dawei Duan, Hongzhong Ma, Yan Yan, Qifan Yang

**Affiliations:** 1 College of Energy and Electrical Engineering, Hohai University, Nanjing 211100, China; 190206030003@hhu.edu.cn (D.D.); 180406030001@hhu.edu.cn (Y.Y.); 190206030007@hhu.edu.cn (Q.Y.); 2 School of Mechanical and Electrical Engineering, Chuzhou University, Chuzhou 239000, China

**Keywords:** metal particle fault, Hurst exponent, particle swarm optimization with adaptive parameter adjustment, VMD, support vector machine

## Abstract

A diagnosis scheme using the Hurst exponent for metal particle faults in GIL/GIS is proposed to improve the accuracy of classification and identification. First, the diagnosis source signal is the vibration signal generated by the collision of metal particles in the electric field. Then, the signal is processed via variational mode decomposition (VMD) based on particle swarm optimization with adaptive parameter adjustment (APA-PSO). In the end, fault types are classified and identified by an SVM model, whose feature vector is composed of the Hurst exponents of each intrinsic mode function (IMF-H). Extensive experimental data verify the effect of this new scheme. The results exhibit that the classification performance of SVM is significantly improved by the new feature vector. Furthermore, the VMD based on APA-PSO with adaptive parameter adjustment can effectively enhance the decomposition quality.

## 1. Introduction

The metal particle contamination within a gas-insulated line (GIL) and gas-insulated switchgear (GIS) is the main threat to their insulation [1,2,3]. Accurate identification of the size and quantity of metal particles can not only provide effective early warning of the severity of equipment fault but also provide data reference for later disassembly and maintenance, thereby ensuring the safe operation of GIL/GIS.

At present, domestic and foreign research about the metal particle fault in GIL/GIS is mainly focused on analyzing the difference between the metal particle fault and other mechanical faults, and only a few scholars have undertaken research involving the identification and diagnosis of the size and quantity of metal particle faults [4,5,6,7]. In Reference [6], Wu et al. corrected and expanded the estimated equations derived by L. E. Lundgarrd and extracted the particle properties based on acoustic amplitude-flight time (AAFT) pattern to estimate the mass, maximum charge and flight height of metal particles in the GIL. Validation experiments indicated that the error of the estimation results fluctuated significantly, which could reach 45.72%. In Reference [7], Zhang et al. collected discharge signals of linear metal particles that were detected by the conventional pulse current detector and ultrasonic detector and then compared the maximum apparent discharge volume and ultrasonic pulse frequency of linear metal particles with the simulation results to estimate the size of linear metal particles. However, the experimental value of the apparent discharge quantity of particles was quite different from the simulated value, and the proposed size estimation algorithm had not been experimentally verified.

Traditional metal particle fault identification is mainly achieved by using ultrasonic amplitude, collision frequency and apparent discharge quantity from the original signal as the feature vectors of particle fault identification [8,9,10]. However, it may cause overlapping, confusion and missing of fault features, which can result in long diagnosis time and low diagnosis accuracy, due to the problems of airgap discharge and random change of collision angle when particles are moving in the electric field. Therefore, it is necessary to select appropriate fault diagnosis methods, such as the model-based, signal-based and learning-based methods [11,12], for improving the accuracy of metal particle fault diagnosis.

When the electric force on the charged particle overcomes the gravitational force and the drag force, the particle lifts off and collides with the enclosure or the insulator inside GIL/GIS. The vibration signal due to the direct collision is more sensitive to metal particles than other available partial discharge signals. In addition, the vibration signal contained abundant and dynamic information, which renders them fit to be used for metal particle fault diagnosis. 

Generally, for vibration-based fault diagnosis, features are often extracted by the signal analysis methods based on modal decomposition, such as wavelet transform [13,14], empirical mode decomposition (EMD) [15], and variational mode decomposition (VMD) [16]. VMD can accurately separate the harmonic components of nonstationary vibration signals and reduce the impact of non-Gaussian impulsive noise. Compared with VMD, EMD lacks a rigorous mathematical theoretical basis and may lead to model confusion. In addition, the diagnosis result of SVM in Reference [17] exhibits that VMD feature extraction outperforms wavelet transform on performance. To sum up, VMD is more applicable for analyzing fault vibration signals in fault detection.

The motion of metal particles in the GIL/GIS cavity is similar to Brownian motion, with a substantial degree of freedom and randomness. As a result, it is almost impossible to illustrate by feature vectors deterministically [18,19]. However, this motion can be regarded as an atypical fractal model due to self-similarity and long-range correlation characteristics. Recently, the Hurst exponent served as a core parameter to uncover multifractality buried in nonlinear and nonstationary vibration signals. It is commonly computed by typical methods, such as the detrended fluctuation analysis (DFA) method, wavelet transformation method, and R/S method [20,21,22,23,24]. In addition, the effectiveness of using the Hurst exponent for fault diagnosis has been investigated in the published research [25,26].

Several algorithms have been developed to diagnose fault vibration signals, such as k-nearest neighbors (KNN), support vector machine (SVM), random forest (RF), decision tree (DT) and convolutional neural network (CNN) [27,28,29,30,31,32]. Among these methods, SVM is good at handling small datasets and nonlinear signals in fault classification tasks. Because the vibration data of metal particle faults are hard to obtain, only a few samples are available for training. Thus, the SVM classifier is more suitable for this diagnosis task.

To solve the problem of poor output results due to improper parameter settings of VMD, in this study, the particle swarm optimization with adaptive parameter adjustment (APA-PSO) will be applied for the self-selection of the VMD model parameters. The best parameter combination of the penalty parameter *β* and the number of subcomponents *K* are obtained, which is used to optimize the VMD algorithm. Then, VMD, with optimized parameters, handles the fault vibration signals. The Hurst exponent of each intrinsic mode function (IMF-H) is calculated as the fault eigenvalue. Moreover, SVM is used to classify metal particle faults. According to the experimental analysis results, the effective fault diagnosis method that is a combination of APA-PSO-VMD, IMF-H, and SVM can improve the identification accuracy of a metal particle fault.

The rest of this paper is organized as follows. In Section 2, the basic theory of VMD and APA-PSO is introduced, and the parameter optimization process of VMD is described. In Section 3, the proposed optimization method is verified by the experimental data analysis and comparison, and the theory and meaning of Hurst exponent for vibration signal are introduced. In Section 4, the vibration signals of metal particle faults are processed by the approach proposed in this paper, and SVM realizes the classification. Furthermore, contrastive analysis among the different methods is conducted in Section 4. Finally, the conclusions are summarized in Section 5.

## 2. Related Algorithm

### 2.1. Variational Mode Decomposition

VMD is a new method of signal decomposition based on one-dimensional Hilbert transform, Gauss smooth demodulation and alternate direction method of multipliers. It decomposes the original signal into several mode components *u_i_* with a specific bandwidth and defines *u_i_* as the amplitude modulation-frequency modulation (AM-FM) signal; thus, the expression is given as follows:

(1) $$u_{i}\left(t\right)=A_{i}(t)\cos(\phi_{i}(t))$$

where i∈{1,…,K}, *A_i_*(*t*) is an envelope function, and *A_i_*(*t*) ≥ 0; $\phi_{i}(t)$ is a nondecreasing function, and ${\phi^{\prime }}_{i}(t)\geq 0$. Moreover, the change of *A_i_*(*t*) and the instantaneous frequency ${\phi^{\prime }}_{i}(t)$ are much slower than the phase $\phi_{i}(t)$.

The Hilbert transform is applied to the modal function *u_i_*(*t*) to construct the analytic signal and then obtain its one-sided spectrum:

(2) $$\left[\delta (t)+j/(\pi t)\right]u_{i}(t)$$

where *δ*(*t*) indicates a pulse function.

The exponential function $e^{-j\omega_{i}t}$ of the center frequency revises the one-sided frequency spectrum of each IMF component to the corresponding fundamental frequency spectrum:

(3) $$\left[\left(\delta (t)+j/(\pi t))u_{i}(t\right)\right]e^{-j\omega_{i}t}$$

The bandwidth of each IMF component is calculated through Gauss smooth demodulation to establish a constrained variational model:

(4) $$\{\begin{array}{l} \underset{\left\{u_{i}\right\},\left\{\omega_{i}\right\}}{\min}\left\{\sum_{i}{\left\|\partial_{t}\left[\left(\delta (t)+j/(\pi t))u_{i}(t\right)\right]e^{-j\omega_{i}t}\right\|}_{2}^{2}\right\}\\ s.t.\sum_{i}u_{i}=m \end{array}$$

where {*u_i_*} = {*u*_1_, …, *u_K_*} indicates the *K* model components; {*w_i_*} = {*w*_1_, …, *w_K_*} denotes the center frequencies.

In order to ensure the accuracy of signal reconstruction and the strictness of constraint conditions, the unconstrained variational problem can be addressed with a Lagrangian multiplier *λ*(*t*) and quadratic penalty parameter *β*, and the augmented Lagrange function is given as follows:

(5) $$\begin{array}{l} L\left(\left\{u_{i}\right\},\left\{\omega_{i}\right\},\lambda \right)=\beta \sum_{i}{\left\|\partial_{t}\left[\left(\delta (t)+j/(\pi t))u_{i}(t\right)\right]e^{-j\omega_{i}t}\right\|}_{2}^{2}\\ \;\;\;\;\;\;\;\;\;+\langle \lambda \left(t\right),m\left(t\right)-\sum_{i}u_{i}\left(t\right)\rangle +{\left\|m\left(t\right)-\sum_{i}u_{i}\left(t\right)\right\|}_{2}^{2} \end{array}$$

where *m*(*t*) indicates the original signal.

The alternate direction method of multipliers (ADMM) has been applied in this paper to iteratively update $u_{i}^{n+1}$, $\omega_{i}^{n+1}$ and $\lambda_{}^{n+1}$. The optimal solution of the constrained variational model can be obtained by searching the saddle point through the Formula (5). The following VMD iterative operation in the present study is as follows:

(1) Initialize $\left\{u_{i}^{1}\right\}$, $\left\{\omega_{i}^{1}\right\}$, *λ*^1^, *n* = 0.
(2) Update $u_{i}$ and $\omega_{i}$ according to the following formula by using the Parseval/Plancherel Fourier isometric transform:
  (6) $${\hat{u}}_{i}^{n+1}\left(\omega \right)=\frac{\hat{m}\left(\omega \right)-\sum_{i}{\hat{u}}_{i}\left(\omega \right)+\frac{\hat{\lambda }\left(\omega \right)}{2}}{1+2\beta {\left(\omega -\omega_{i}\right)}^{2}}$$
  (7) $$\omega_{i}^{n+1}=\frac{\int_{0}^{\infty }\omega {\left|{\hat{u}}_{i}\left(\omega \right)\right|}^{2}d\omega }{\int_{0}^{\infty }{\left|{\hat{u}}_{i}\left(\omega \right)\right|}^{2}d\omega }$$
  where ${\hat{u}}_{i}^{n+1}(\omega )$ is calculated through a Wiener filter with a prior 1/(*ω* − *ω_i_*)^2^, and the time domain is calculated as the real part of ${\hat{u}}_{i}^{n+1}(\omega )$ by Fourier transform; where $\omega_{i}^{n+1}$ is the corresponding central frequency of current modal component.
(3) Update *λ* according to the following formula:
  (8) $$\lambda^{n+1}=\lambda^{n}+\tau \left(m-\sum_{i}u_{i}^{n+1}\right)$$
(4) Settle the iteration terminated criteria as the following formula:
  (9) $$\sum_{i}{\left\|u_{i}^{n+1}-u_{i}^{n}\right\|}_{2}^{2}/{\left\|u_{i}^{n}\right\|}_{2}^{2}<\varepsilon$$
  where *ε* is a given accuracy threshold. Repeat step (2) until the function converges, which is to satisfy the condition of Formula (9).

### 2.2. Particle Swarm Optimization with Adaptive Parameter Adjustment-Based VMD (APA-PSO-VMD)

In the traditional algorithm of VMD, the user needs to set the penalty parameter *β* and the number of the components *K* before processing the signal because of the theory limitation [33,34,35]. The improper selection of the two parameters will result in some unacceptable mode compositions. Thus, selecting the optimal parameter group of the penalty parameter and the number of the components is the key to accurately extracting the fault information.

The particle swarm optimization (PSO) algorithm is a population-based stochastic approach for solving global optimization problems, which is suitable for the optimal selection of parameters in consideration of its simple mechanism, easy adjustment, few control parameters and wide search range [36]. On the strength of the search strategy of the PSO algorithm, each particle searches for a better position by changing its migration velocity according to rules inspired by birds’ foraging behavior. To solve the problem of falling into the local optimum, the adaptive adjustment strategy of inertia weight and learning factor is proposed. It can reconstruct the particles with weak evolutionary ability, and then balance the local search and global search capabilities, so the PSO algorithm can jump out of the local optimal and obtain the approximate solution of the global optimal [37].

Particle evolution ability and population evolution ability are defined as follows:

(10) $$E_{h}^{n}=\frac{\delta_{w}^{n}-\delta_{h}^{n}}{\delta_{w}^{n}-\delta_{b}^{n}}$$

(11) $$E_{g}^{n}=\delta_{g}^{n}-\delta_{g}^{n-1}$$

where $\delta_{h}^{n}$ indicates the fitness of the *h*-th particle of the *n*-th generation; $\delta_{g}^{n}$ indicates the fitness of the optimal particle in the history of the *n*-th generation group; $\delta_{w}^{n}$ and $\delta_{b}^{n}$ indicate the worst and best fitness of all particles in the *n*-th generation, respectively.

The population evolution rate of particles is defined as follows:

(12) $$X_{h}^{n}=1/\sqrt{E_{g}^{n2}+E_{h}^{n2}+1}$$

The independent adaptive inertia weight for each particle is settled to ensure the diversity of particles and the efficiency of the search as follows:

(13) $$\varphi_{h}^{n+1}=\varphi_{\mathrm{init}}-\left(\varphi_{\mathrm{init}}-\varphi_{\mathrm{end}}\right)\times X_{h}^{n+1}$$

where *φ*_init_ is the initial weight; *φ*_end_ is the final weight.

The learning factors *c*_1_ and *c*_2_ are settled as the decreasing function and the increasing function, respectively. We adjust the learning factor of each particle according to the evolution rate so it can not only ensure the global search capability at the initial stage of the iteration but also can strengthen the local accurate search capability at the later stage of the iteration. The proposed adaptive formula of the learning factor is expressed as:

(14) $$\{\begin{array}{l} c_{1h}^{n+1}=c_{1\max}-\frac{c_{1\max}\sin\left(\pi X_{h}^{n+1}/2\right)\times n}{N}\\ c_{2h}^{n+1}=c_{2\min}+\frac{c_{2\min}\sin\left(\pi X_{h}^{n+1}/2\right)\times n}{N} \end{array}$$

where *c*_1max_ is the maximum of the learning factor; *c*_2min_ is the minimum of the learning factor; *N* is the maximum number of iterations.

*P*_1_ particles with weaker evolutionary ability are selected according to the minimum mean value of envelope entropy as the fitness value and are reconstructed by learning from the remaining *P*_2_ particles. Particles are selected according to the following formula:

(15) $$\{\begin{array}{l} P_{1}=round\left(0.8\times P\times \frac{n}{N}\right)\\ P_{2}=P-P_{1} \end{array}$$

where *P* is the total number of the population.

The number of particles that are selected for reconstruction can reach up to 80% of the total population, and the condition for reconstruction is that the random value generated by the *i*-th dimension of each selected particle must be greater than the learning probability *P*_c_. 

**Figure 1:**
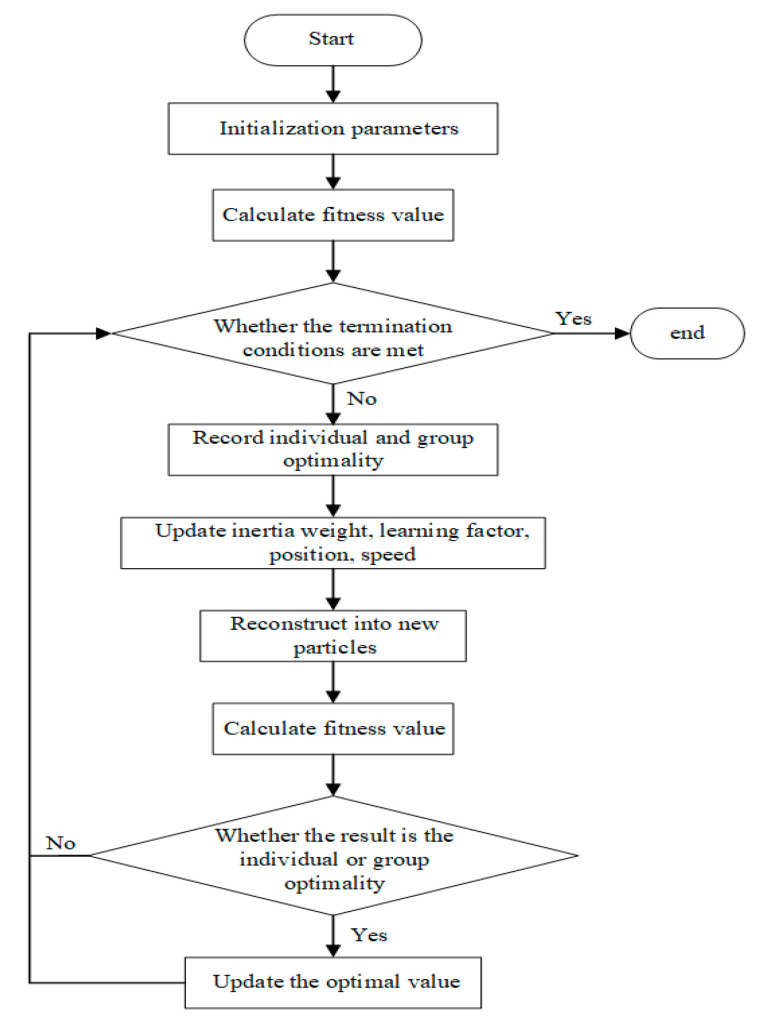
The flow chart of the APA-PSO-VMD method.

The flow chart of the proposed APA-PSO-VMD method is shown in Figure 1.

## 3. Feature Extraction Process of Vibration Signals

### 3.1. Vibration Signals Collection

**Figure 2:**
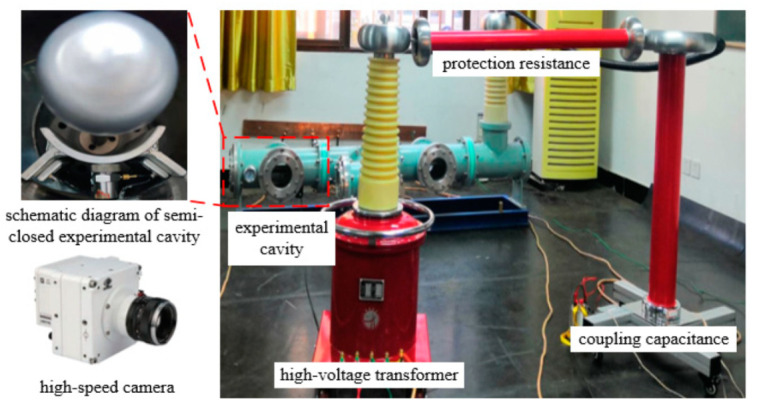
Schematic diagram of the experimental equipment.

The experimental platform is built as shown in Figure 2, which consists of a high-voltage transformer, a protection resistance, a coupling capacitance, an experimental cavity proportional shrunken according to the GIL/GIS prototype, a semi-closed experimental cavity with an inner radius of 45 mm and the central angle of 160°, and a high-speed camera titled VEO 710 L. The electric field structure of the semi-closed experimental cavity within the moving range of metal particles is basically the same as the electric field structure of the fully enclosed experimental cavity at the AC voltage of 50 kV. It can be considered that this experimental platform is the closest equivalent to the actual GIL/GIS operating platform in macroscopic view [38,39].

**Figure 3:**
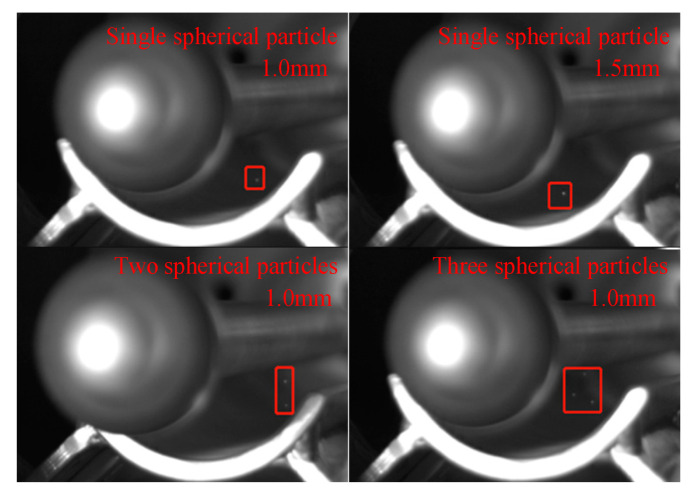
Movement images of spherical particles.

In order to simulate the four types of metal particle faults in the GIL/GIS equipment: single spherical particle (1.0 mm), single spherical particle (1.5 mm), two spherical particles (1.0 mm) and three spherical particles (1.0 mm) are, respectively, placed at the middle of the bottom of the semi-closed experimental cavity. Figure 3 shows the movement images of spherical particles taken by a high-speed camera.

**Figure 4:**
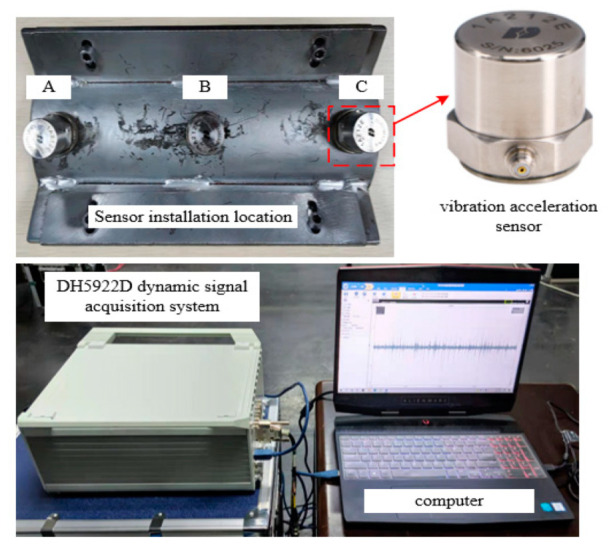
Schematic diagram of acquisition equipment.

The data acquisition system is shown in Figure 4, three vibration acceleration sensors (1A212E) are placed at the bottom of the semi-closed cavity with equal distances and marked A, B, C in sequence. The sensors have the characteristics of high resolution, wide measuring range and strong anti-interference ability. The specific parameters of the sensor (1A212E) are shown in Table 1. Then, three groups of vibration signal are collected according to the DH5922D dynamic signal acquisition system with a sampling frequency of 20 kHz. Furthermore, the accuracy and completeness of the collected vibration signals can be guaranteed by referring to the movement images of metal particles.

**Figure 5:**
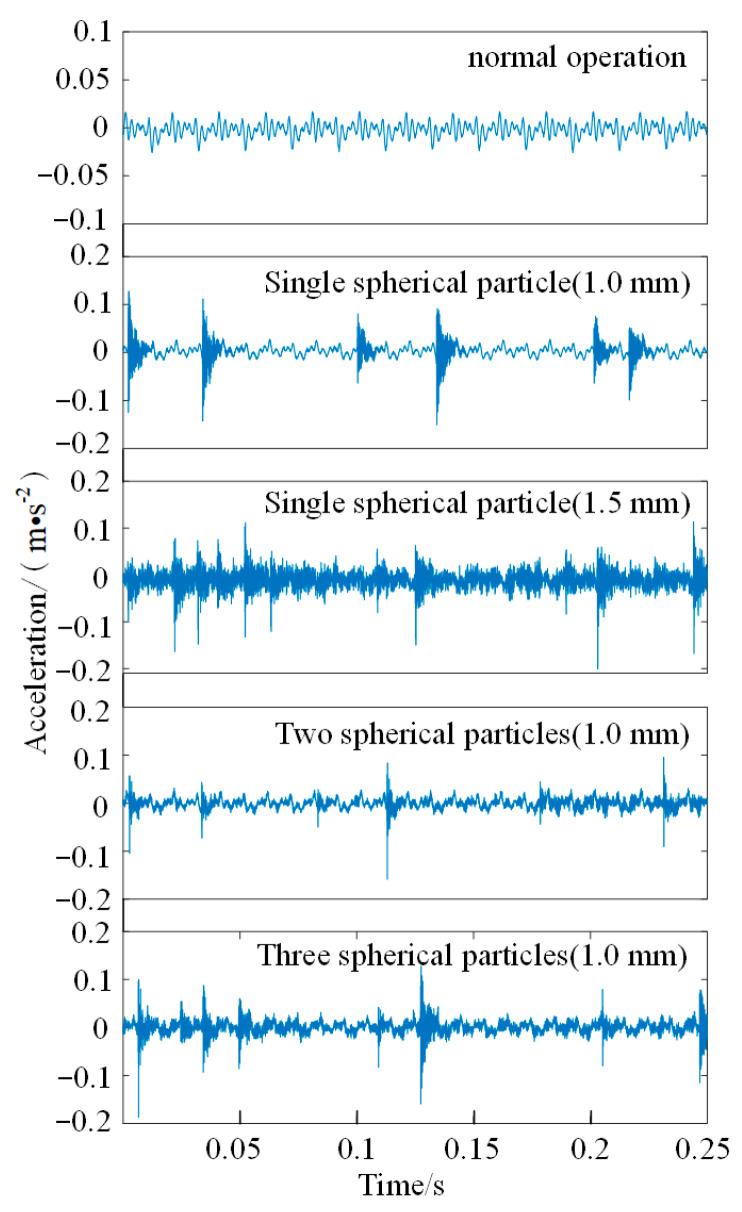
Vibration signals of different operating states.

At the beginning of the experiment, the metal particles are placed in the middle of a semi-closed cavity, and the vibration signals of different operating states collected by the dynamic signal acquisition system according to sensor B are shown in Figure 5. The vibration acceleration sensor can only collect the subtle corona sound in the experimental cavity under normal operating state of GIL/GIS. The collected signals change regularly, and the maximum amplitude can only reach 0.025 m/s^2^. When the metal particle fault occurs, the amplitude and frequency of the collision signal will change due to the size and quantity of the particles; in addition, the micro-discharge and gap discharge caused by particle movement will also aggravate the background noise of the signal. Figure 5 also indicates that the maximum amplitude above the vibration signals and the collision frequency of particle movement are somewhat different. However, the amplitude and frequency of different vibration signals will not show regular changes due to the sinusoidal change of AC voltage, the randomness of collision and the interaction between particles. Therefore, it is impossible to accurately classify the metal particle faults only based on the collision frequency, the amplitude and the frequency of vibration signals.

### 3.2. Vibration Signal Modal Decomposition

In order to prove the superiority of the APA-PSO-VMD method for the decomposition effect of metal particle fault signals, it is compared with the decomposition effects of the EMD and the original VMD method, respectively.

**Figure 6:**
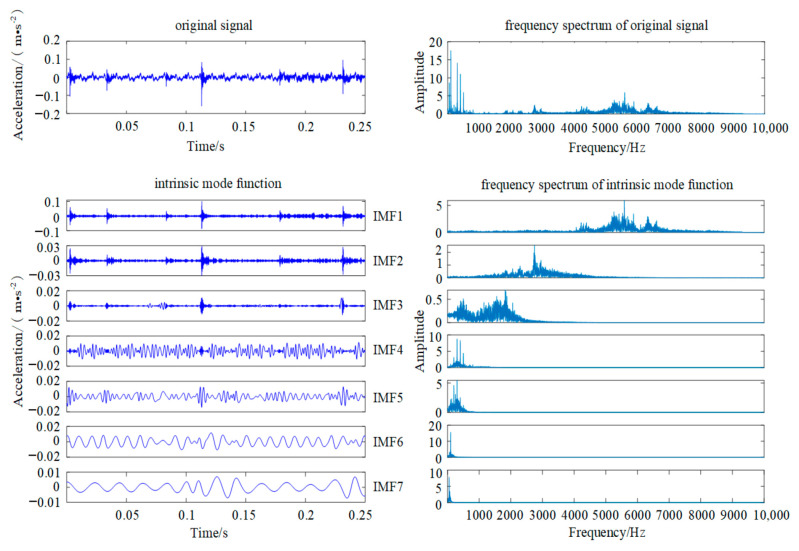
EMD decomposition diagram.

EMD is used to decompose the fault vibration signal of two spherical particles, as shown in Figure 5. The result of the envelope spectrum analysis demonstrates that it seriously affects the quality of the extracted fault features due to the problems of model confusion, over-decomposed (decomposing a lot of noise components) and under-decomposed (missing part of the main frequency components), which is presented in Figure 6.

**Figure 7:**
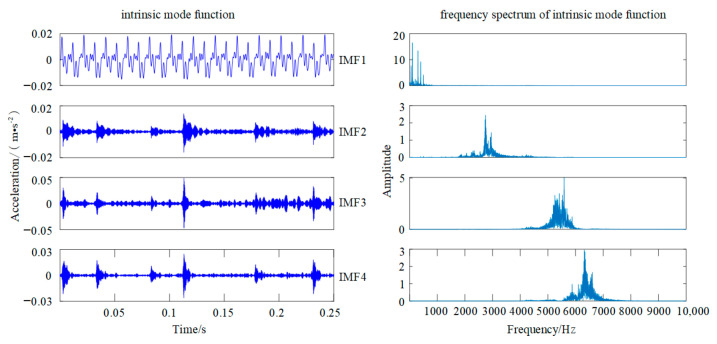
Original VMD decomposition diagram.

Similarly, the original VMD method is used to decompose the same segment of fault signal. The number of components and the penalty parameter are empirically set at 4 and 2000, respectively. As shown in Figure 7, the original VMD method solves the problem of modal confusion in the EMD algorithm and improves decomposition accuracy and quality of feature extraction. However, the problem of under-decomposed still exists in the original VMD algorithm, which may affect the accuracy of fault classification.

**Figure 8:**
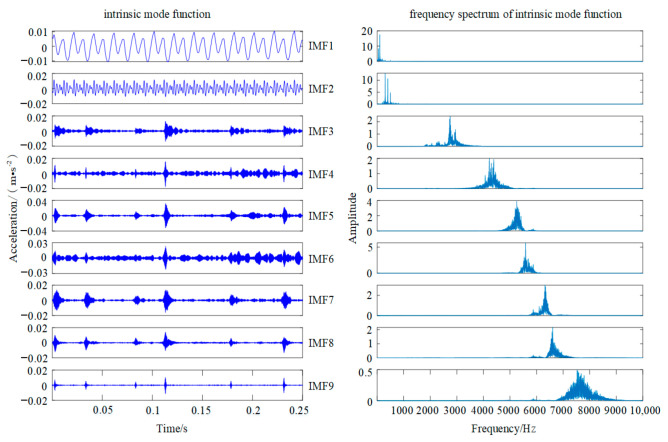
APA-PSO-VMD decomposition diagram.

The APA-PSO-VMD algorithm is applied to decompose the same fault signal. In this study, the population size is 20, the maximum number of iterations is 20, the initial weight and the final weight are 0.9 and 0.4, respectively, the maximum of learning factor and the minimum of learning factor are 2 and 0.7, respectively, and the learning probability is 0.8. The number of components and the penalty parameter optimized by the APA-PSO search algorithm is a collection of (9, 1803), which means that the selected particle fault signal can be decomposed into nine sets of subcomponents, and the APA-PSO-VMD decomposition diagram is shown in Figure 8.

As shown in Figure 8, the APA-PSO-VMD algorithm can solve the problems of modal confusion and under-decompose better than the EMD method and the original VMD method. Furthermore, almost all the main frequency bands with obvious characteristics are decomposed independently. Thus, the decomposition result of APA-PSO-VMD indicates that the identification accuracy of fault feature frequency is improved compared with the above two methods, which can provide assistance for the feature selection and classification of metal particle faults.

### 3.3. Feature Vectors of Vibration Signal

The Hurst exponent was proposed by British hydrologist H.E. Hurst in the middle of the 20th century, which can be used as a core parameter to characterize the fractal characteristics of fault signals. Currently, there are many methods used to compute the Hurst exponent, and the typical R/S method is used in this study. 

For a given time series {*Y*_1_, *Y*_2_, …, *Y_q_*} of vibration signals, the average value *e*(*v*) and the variance *S*(*v*) of each subinterval can be respectively calculated considering a number of subintervals each of size *v*, as shown in the following formulas:

(16) $$e\left(v\right)=\frac{1}{v}\sum_{q=1}^{v}Y_{q}$$

(17) $$S\left(v\right)=\sqrt{\frac{1}{v-1}\sum_{q=1}^{v}{\left(Y_{q}-e\left(v\right)\right)}^{2}}$$

The cumulative deviation *Z*(*q*,*i*) and the range *R*(*v*) are defined as:

(18) $$Z\left(q,i\right)=\sum_{q=1}^{v}\left(Y_{q}-e\left(v\right)\right)$$

(19) $$R\left(v\right)=\underset{1\leq i\leq v}{\max}Z\left(q,i\right)-\underset{1\leq i\leq v}{\min}Z\left(q,i\right)$$

Then, the dimensionless ratio *R*(*v*)/*S*(*v*) is introduced, and rescale *R_s_*(*v*) is calculated for each subinterval as in the following formula:

(20) $$R_{s}\left(v\right)=\frac{R\left(v\right)}{S\left(v\right)}$$

In order to compare the vibration signals of different states, the logarithmic equation with the Hurst exponent is obtained as:

(21) $$\mathrm{lg}\left(R_{s}\left(v\right)\right)=\mathrm{lg}b+H\mathrm{lg}v$$

where *b* is a constant; *H* is the Hurst exponent, and the value of the Hurst exponent can be obtained by the slope of the linear regression of (lg*v*, lg*R_s_*(*v*)).

The meaning of the Hurst exponent in the vibration signal can be explained based on a series of studies:

(1) When 0 < *H* < 0.5, it shows that the time series of vibration signal presents the characteristics of inverse long-term correlation and independence, and the future overall trend is contrary to the past, which is to say, the past is increasing, and the future is decreasing. The vibration signal, therefore, has strong variability and mutability due to the ongoing reversion.
(2) When *H* = 0.5, it shows that the time series of the vibration signal is purely random, and the variables will not affect the future completely.
(3) When 0.5 < *H* < 1, it indicates that the time series of the vibration signal presents the characteristics of positive long-term correlation and independence, and the general trend of the future will inherit the general trend of the past; thus, the process is sustainable. In addition, the future trend of vibration signals can be predicted when the Hurst exponent is 1.

**Figure 9:**
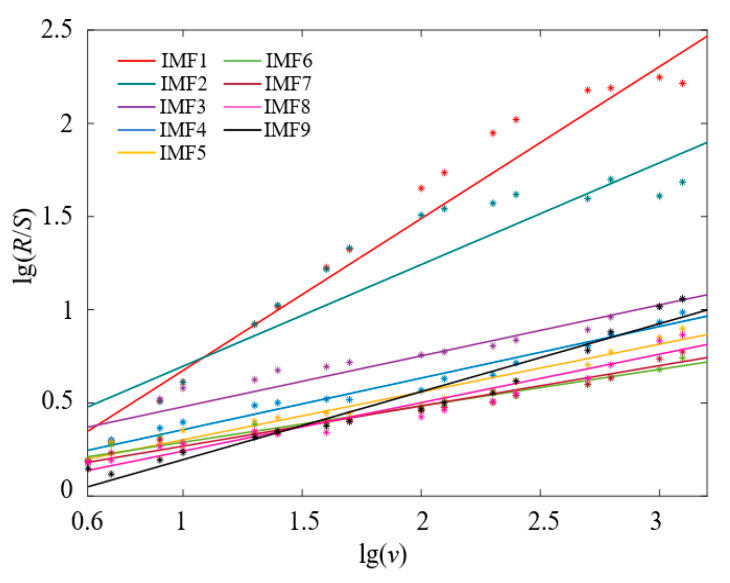
Hurst exponent diagram for intrinsic mode function.

In order to reflect the dynamic characteristics of the metal particle fault signal, the APA-PSO-VMD algorithm is applied to decompose the vibration signal and then calculate the Hurst index of each IMF component (IMF-H), which is used to be the feature vector of metal particle fault classification. The Hurst exponent diagram for the intrinsic mode function of the fault vibration signals of two spherical particles in Figure 5 is shown in Figure 9, and the calculated values of the Hurst exponent are shown in Table 2.

Figure 9 and Table 2 indicate that the time series of the metal particle fault signal is not a purely random process, and the information contained in the vibration signal measured on site is not independent; furthermore, the dynamic change of metal particle movement in GIL/GIS has a measurable long-term memory. The values of IMF1-H and IMF2-H are all greater than 0.5, which indicate that the IMF1 and the IMF2 present the characteristics of positive long-term correlation and persistence. Based on the description of the Hurst exponent in the normal operating state of equipment in Reference [40], it can be concluded that the IMF1 and the IMF2 decomposed by VMD contained the feature information of a normal state signal. Relatively, the Hurst exponents of IMF3 to IMF9 show that the corresponding components contain the feature information of the vibration signals due to the metal particle collision and present the characteristics of inverse long-term correlation, strong variability and mutability.

## 4. Fault Diagnosis and Analysis

### 4.1. Fault Diagnosis Steps

For the problems of difficult acquisition of metal particle fault data in GIL/GIS and the low accuracy of fault diagnosis, APA-PSO-VMD and SVM are used in this study, which take IMF-H as the feature vector for fault identification and classification. The specific steps are as follows:

(1) The vibration signals of the normal state and four types of metal particle fault states are collected by the dynamic signal acquisition system with a sampling frequency of 20 kHz. For each state, 60 groups of sample data are collected, wherein each data group consists of 5000 samples.
(2) The best parameter combination (*β*_1_, *K*_1_) of the VMD algorithm for each state is obtained by using the particle swarm optimization with the adaptive parameter adjustment method. In this method, the population size is *N* = 20, the maximum number of iterations is *τ* = 20, the initial weight is *φ*_init_ = 0.9, the final weight is *φ*_end_ = 0.4, the maximum of learning factor is *c*_1max_ = 2, the minimum of learning factor is *c*_2min_ = 0.7, and the learning probability is *P*_c_ = 0.8.
(3) After the APA-PSO process, the penalty parameter and the number of subcomponents are settled as *β* = *β*_1_ and *K* = *K*_1_. The parameter-optimized VMD method is used to decompose the vibration signals of each state, so as to obtain *K*_1_ intrinsic mode functions and then calculate the Hurst index of each IMF component.
(4) The fault diagnosis model is established by using SVM, which takes IMF-H as the feature vector input. The feature vectors are randomly divided into five sets according to the fivefold cross validation, of which four sets are selected as training samples set to train the SVM diagnostic model, and the one remaining set is selected as a test sample.

### 4.2. Diagnosis Results Analysis

For the collected vibration signals of GIL/GIS in a normal operation state and particle fault states, the best parameter combination (*β*_1_, *K*_1_) of the VMD algorithm for each group signal is obtained, as shown in Table 3.

It can be seen from Table 3 that the *K*_1_ value of the normal state is quite different with fault signals. Moreover, the normal state and particle fault states can be effectively distinguished according to the time domain signals because the vibration signals of the normal state change regularly and the maximum amplitude can only reach 0.025 m/s^2^. Therefore, just the identification and classification of different metal particle faults are concerned in the fault diagnosis. The corresponding optimal parameter combination of each group signal is substituted into the VMD algorithm, and the Hurst index of each IMF component decomposed by VMD is calculated. In order to compare the feature vectors of different faults and improve the accuracy of fault diagnosis, the first nine IMF-H values of the vibration signals collected by three sensors at the same time are all used as the corresponding feature vectors, which are shown in Table 4. In Table 4, single particle fault (1.0 mm), single particle fault (1.5 mm), two particles fault (1.0 mm), and three particles fault (1.0 mm) are marked as Fault A, Fault B, Fault C, and Fault D, respectively.

**Figure 10:**
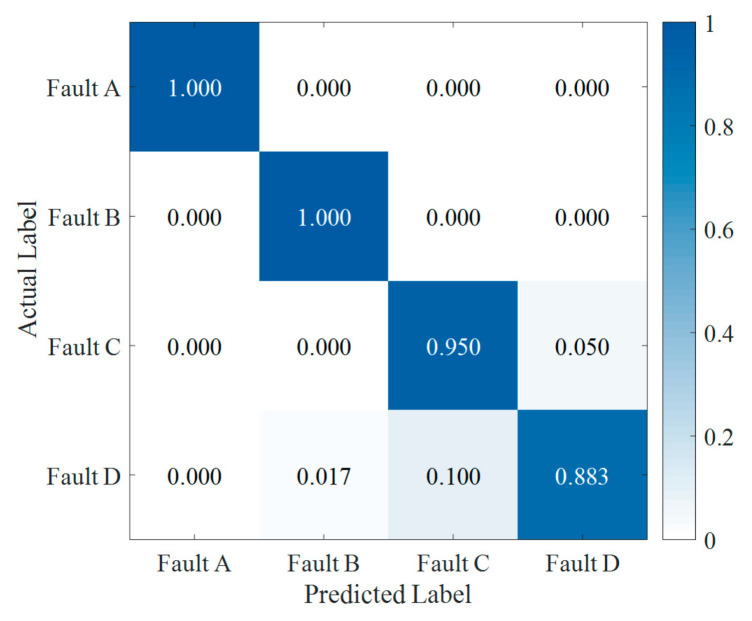
Confusion matrix of the classification results using SVM.

The fault feature vectors, namely the IMF-H of fault states, are input into the SVM diagnostic model in the fault diagnosis process. For each metal particle fault, a total of 60 groups of datasets are regarded as feature vectors, and each group is composed of 27 IMF-H. Fivefold cross validation evaluates the performance of the classification model in this task. The dataset is randomly split into five sets. Four sets are selected to train an SVM diagnostic model, and the remaining set serves as a test set. Using the confusion matrix, Figure 10 manifests the fault diagnosis result of the four types of faults, whose accuracies are 100%, 100%, 95%, and 88.3%, respectively. Thus, the average classification accuracy of the method proposed in this paper can reach 95.8%.

In order to evaluate the performance of the proposed diagnosis scheme in this study, a comprehensive comparison is made with four feature vectors and three conventional fault detection methods. Accuracy and F1 score are used as evaluation metrics for evaluating the robustness and generalization properties of fault diagnosis models. The combination of these two metrics can adequately display the diagnosis results and evaluate the classification performance of the corresponding methods. Table 5 illustrates the comparison results of different diagnosis models within average fivefold cross validation.

According to Table 5, based on SVM, the proposed feature vector outperforms all other feature vectors in accurately classifying metal particle faults. Our framework is 22% more accurate than conventional approaches based on EMD and SVM. In contrast, the (*f*, *V*)-SVM approach cannot accurately extract features of fault signals, leading to the lowest accuracy, i.e., 49.6%. Furthermore, using the same feature vector, the proposed approach performs better in accuracy than other learning-based methods, such as KNN, RF, and DT. In conclusion, in the case of a small amount of experimental data, the classification performance based on SVM is significantly better than that based on KNN, RF, and DT.

Moreover, the approaches using the proposed feature vector reach an F1 score of more than 0.9, while the highest F1 score of other approaches is only 0.892. With (*f*, *V*) and original signal features, the F1 scores of diagnosis models are, respectively, 0.503 and 0.663, which are lower than that of models with the IMF-H feature. In summary, the proposed approach can effectively extract the fault features from the vibration signals of metal particle fault, with better robustness and generalization properties than other existing approaches. Furthermore, the new feature vector proposed in this study can provide new ideas for metal particle fault diagnosis and related research.

## 5. Discussion

This paper aims to present a quantitative and pragmatic diagnosis of the metal particle faults within GIL/GIS. The IMF-H is proposed as a new feature vector for the vibration signals of metal particle fault, and the fault diagnosis method proposed is based on the APA-PSO-VMD method and SVM. Moreover, comparative analysis based on the experimental data verifies the effectiveness and superiority of the proposed method.

(1) The variational mode decomposition method based on the particle swarm optimization with adaptive parameter adjustment is applied to decompose and reconstruct the original vibration signal of metal particle faults, which can better display the fault characteristic information than the original VMD method and the EMD method, therefore, improving the quality of extracted fault feature.
(2) The analysis results show that the IMF-H based on APA-PSO-VMD can accurately classify metal particle faults, outperforming all other feature vectors in terms of identification accuracy. Furthermore, for the metal particle fault with a small dataset, the classification performance based on SVM is significantly better than that based on KNN, RF, and DT. Thus, the proposed approach can effectively improve the robustness and generalization properties of the fault diagnosis model.
(3) The IMF-H is innovatively used as an effective feature vector for metal particle fault identification, which can provide new ideas for metal particle fault diagnosis and related research.

Future work will focus on a deep learning-based diagnosis strategy for detecting metal particle faults of GIL/GIS.

## Figures and Tables

**Table 1:** Specific parameters of the sensor 1A212E.

Dynamic Indicator	Value
Axial sensitivity	49.92/mV/m/s^2^
Maximum lateral sensitivity	<5%
Frequency response	0.2~4000 Hz
Resolution	0.00005 g
Electromagnetic sensitivity	5 g/T

**Table 2:** IMF-H exponent numerical value of the vibration signal.

Modal Component	Hurst Exponent	Modal Component	Hurst Exponent
IMF1	0.8153	IMF6	0.1995
IMF2	0.5458	IMF7	0.2161
IMF3	0.2732	IMF8	0.2600
IMF4	0.2769	IMF9	0.3648
IMF5	0.2565		

**Table 3:** Best parameter combination of vibration signals.

Operating Status	Penalty Parameter *β*_1_	Number of Subcomponents *K*_1_
Normal operation	1159	2
Single particle fault (1.0 mm)	1654	10
Single particle fault (1.5 mm)	927	9
Two particles fault (1.0 mm)	1803	9
Three particles fault (1.0 mm)	1770	11

**Table 4:** IMF-H exponent numerical value of metal particle faults.

Signal Source	Fault Category	IMF1-H	IMF2-H	IMF3-H	IMF4-H	IMF5-H	IMF6-H	IMF7-H	IMF8-H	IMF9-H
Sensor A	Fault A	0.5038	0.8605	0.3479	0.3292	0.2739	0.2719	0.272	0.2225	0.2429
	Fault B	0.7337	0.2458	0.2041	0.1782	0.2127	0.1676	0.1831	0.1576	0.1459
	Fault C	0.8146	0.5309	0.2458	0.1679	0.167	0.2713	0.2221	0.2548	0.2391
	Fault D	0.7441	0.3179	0.235	0.2402	0.1605	0.1541	0.2093	0.1923	0.1982
Sensor B	Fault A	0.8277	0.5362	0.3448	0.3473	0.2696	0.2254	0.2767	0.2657	0.2983
	Fault B	0.8251	0.3519	0.2313	0.1735	0.1540	0.1896	0.2510	0.2437	0.3188
	Fault C	0.8153	0.5458	0.2732	0.2769	0.2565	0.1995	0.2161	0.2600	0.3648
	Fault D	0.7970	0.4007	0.3060	0.2594	0.2430	0.2188	0.2414	0.2542	0.3528
Sensor C	Fault A	0.8185	0.525	0.2674	0.3144	0.2435	0.2668	0.2227	0.2362	0.2576
	Fault B	0.8391	0.3182	0.2605	0.1739	0.1498	0.1616	0.2076	0.211	0.2125
	Fault C	0.807	0.5002	0.342	0.3303	0.2163	0.2487	0.2712	0.2806	0.258
	Fault D	0.7767	0.317	0.2362	0.3375	0.196	0.2279	0.2043	0.2145	0.235

**Table 5:** The results of different diagnosis models for metal particle fault.

Feature Vector	Method	Accuracy	F1 Score
IMF-H based on VMD (*b* = 2000, *K* = 4)	SVM	0.892	0.892
IMF-H based on EMD	SVM	0.738	0.739
Particle collision frequency *f* and maximum collision amplitude *V*	SVM	0.496	0.503
Original vibration signal	SVM	0.654	0.663
IMF-H based on APA-PSO-VMD	KNN	0.913	0.912
IMF-H based on APA-PSO-VMD	RF	0.946	0.945
IMF-H based on APA-PSO-VMD	DT	0.917	0.907
**IMF-H based on ** **APA-PSO-VMD**	**SVM**	**0.958**	**0.958**

## Data Availability

Not applicable.
